# D-pFoodReQ: Answer-Frequency-Aware Debiasing for Constrained Knowledge-Base Question Answering in Food Recommendation

**DOI:** 10.3390/foods15193418

**Published:** 2026-09-24

**Authors:** Zhifang Liu, Wenchao Liu, Guorui Sheng, Yancun Yang, Weiqing Min, Shuqiang Jiang

**Affiliations:** 1 School of Computer Science and Artificial Intelligence, Ludong University, Yantai 264025, China; liuzhifang@m.ldu.edu.cn (Z.L.); liuwenchao@ldu.edu.cn (W.L.); yangyancun@ldu.edu.cn (Y.Y.); 2 State Key Laboratory of AI Safety, Institute of Computing Technology, Chinese Academy of Sciences, Beijing 100190, China; minweiqing@ict.ac.cn (W.M.); sqjiang@ict.ac.cn (S.J.); 3 School of Computer Science and Technology, University of Chinese Academy of Sciences, Beijing 100190, China

**Keywords:** food recommendation, knowledge-base question answering, answer frequency bias, counterfactual intervention, knowledge graph

## Abstract

Food recommendation systems must satisfy dietary preferences and nutritional, health, and ingredient constraints. In constrained food knowledge-base question answering, unequal positive training-answer frequency may influence ranking without necessarily reflecting query–recipe fit. We develop D-pFoodReQ by replacing the pFoodReQ answer ranker with BAMnet-D while retaining its food-knowledge and constraint-processing pipeline. BAMnet-D introduces a separate answer-frequency branch alongside semantic matching, applies candidate-set-relative loss reweighting, and uses a counterfactual-inspired intervention that sets the explicit frequency input to zero during validation and testing. Across three runs using the same protocol on the pFoodReQ benchmark, D-pFoodReQ achieved an F1 score of 62.96 ± 2.06%, compared with 61.33 ± 2.27% for pFoodReQ. Its mean average precision and mean average recall were 66.50 ± 0.54% and 65.38 ± 0.66%, respectively. On 1355 questions for which no gold answer appeared as a positive training label, D-pFoodReQ attained a mean answer-set F1 nearly identical to that of pFoodReQ while achieving higher Recall@5 and NDCG@5. Ablation and output-composition analyses indicated that loss reweighting alone did not account for the full improvement, while retaining the explicit frequency term was associated with a larger share of answers observed as positive training labels. These results support answer-frequency-aware ranking for constrained food knowledge-base question answering.

## 1. Introduction

Digital food systems support food perception, retrieval, recommendation, and dietary monitoring [1]. Within this broader field, food recommendation must account for context and domain knowledge because dietary choices involve nutrition, health requirements, ingredient restrictions, and user preferences [2]. Representative systems illustrate complementary strategies. The PROTEIN AI Advisor combines expert-validated meals with ingredient-appropriateness rules and nutrient-target optimization [3]. FKGM integrates dietary preference and personalized health requirements through a collaborative recipe knowledge graph and multi-task learning [4]. CARE translates dietary requests into structured targets and verifies retrieved recipes against nutrient and ingredient constraints [5]. Together, these studies frame food recommendation as a constrained decision problem that extends beyond preference matching.

The evidence available to such systems is heterogeneous. FoodOn and FoodKG organize food concepts, recipes, ingredients, nutrients, and provenance in machine-readable form [6,7]. Computer-vision methods can estimate nutrient content from food images. For example, DPF-Nutrition combines depth prediction with RGB-D feature fusion for monocular-image nutrition estimation [8]. Ingredient substitution introduces another form of constraint because sensory, functional, nutritional, cultural, and regulatory requirements may need to be considered jointly [9]. These sources provide different kinds of evidence for food selection.

Optimization and language-based systems extend this evidence from individual recipes to meal plans. Reinforcement learning and hybrid optimization have been used to balance user acceptance with nutritional requirements [10,11], while language-model-assisted planning has examined the identification and decomposition of compound ingredients [12]. Across these approaches, encoded constraints can guide a user only when suitable recipes are retrieved and ranked highly enough to be returned. Ranking reliability is therefore a central algorithmic requirement in health-aware and constraint-aware food recommendation.

One way to formulate constrained recipe retrieval is through constrained knowledge-base question answering (KBQA). In this formulation, a dietary request is treated as a question and recipes are ranked as candidate answers, with pFoodReQ serving as a representative framework for this task [13]. It expands a user query with preferences and health guidance. It also augments a food knowledge graph for nutrient comparison and marks positive and negative constraints before ranking candidate recipes. Its bidirectional attentive memory network (BAMnet) answer ranker matches the query with candidate entity types, relation paths, contextual entities, and constraint indicators [14].

The pFoodReQ benchmark constructs constrained questions from FoodKG and health guidance. It records one or more gold answers for each question. Unlike an interaction dataset, it contains no records of impressions, clicks, ratings, or consumption. It nevertheless provides an observable form of uneven supervision: some recipes recur as positive training answers, whereas many receive little or no positive-label support. Such repetition may reflect meaningful regularities in the benchmark, including common recipe types and annotation coverage, but it may also favor candidates independently of how well they match a new question. We refer to this query-independent tendency as answer-frequency bias. It is related to, but distinct from, conventional popularity bias derived from user exposure or interaction [15,16,17].

D-pFoodReQ examines this issue within the answer-ranking stage of pFoodReQ. BAMnet-D retains the original query, candidate-memory, and constraint representations while treating positive training-answer frequency as an additional influence on candidate ranking. Inspired by prior counterfactual and causal approaches to popularity bias in recommendation, we examine the rankings obtained when the direct contribution of this explicitly modeled signal is absent [18,19]. In the supervised constrained food-KBQA setting considered here, this perspective is realized through candidate-set-relative frequency normalization, frequency-aware question weighting, and validation and test rankings formed under the reference condition P=0. Figure 1 summarizes this workflow.

The training-label distribution motivates this control. Figure 2 shows that the training set contains 13,459 gold labels for 4672 questions. These labels cover 9650 distinct recipes. Among them, 6853 recipes occur once and account for 71.02% of the distinct recipes. The maximum frequency is 13. The most frequent 10% and 20% of recipes account for 21.86% and 36.20% of all gold labels. Although this imbalance does not by itself demonstrate biased predictions, it creates unequal positive-label support that may influence answer ranking.

The main contributions are as follows.

- We characterize positive training-answer frequency as an observable indicator of uneven supervision in constrained food KBQA, distinguishing it from popularity derived from user exposure or interaction.
- We develop BAMnet-D, which combines candidate-set-relative answer-frequency modeling with question-level loss reweighting and forms validation and test rankings without the direct contribution of the explicit frequency score.
- We evaluate the method across repeated runs, component variants, answer-frequency regimes, constraint diagnostics, and prediction-set composition. Under the same experimental protocol, D-pFoodReQ obtains higher mean F1, MRR, MAP, MAR, Recall@5, and NDCG@5 than its direct parent model. On questions whose gold answers have zero training frequency, D-pFoodReQ attains a mean answer-set F1 nearly identical to that of pFoodReQ while achieving higher Recall@5 and NDCG@5.

## 2. Related Work

### 2.1. Health-Aware Food Recommendation

Health-aware food recommendation broadly encompasses recipe recommendation, meal planning, nutritional decision support, and conversational dietary guidance. Prior methods can be organized by whether they integrate health evidence into preference learning, encode food requirements as structured knowledge, or optimize complete meals and dietary plans.

Preference- and health-integration methods jointly model what users are likely to choose and what is nutritionally suitable. Yum-me and the model of Yera Toledo et al. incorporate nutritional expectations, restrictions, or nutrient attributes into personalized recommendation [20,21]. Market2Dish adds ingredient and health information to dish retrieval [22]. More recent graph methods represent these relations explicitly. FKGM learns preference and health tasks over a collaborative recipe knowledge graph [4], whereas NRKG connects user nutrition-related preferences with recipes that have related nutritional profiles [23]. MOPI-HFRS further treats preference, personalized healthiness, and nutritional diversity as joint objectives [24]. These methods differ in supervision and architecture, but each places nutritional evidence inside the recommendation decision.

Knowledge-based methods express food requirements through expert rules, ontologies, and graph relations. The PROTEIN AI Advisor separates ingredient-appropriateness verification from nutrient-target meal-plan optimization [3]. FoodOn harmonizes food terminology, while FoodKG connects recipes with ingredients, nutrients, constraints, and provenance [6,7]. ForestFoodKG provides a more recent example of organizing taxonomic and nutritional attributes as a food knowledge graph [25]. CARE combines structured food knowledge with constraint-driven retrieval and verification [5]. KERL and NGQA likewise use graph-structured evidence for recipe selection and nutritional reasoning [26,27]. This line is particularly relevant to constrained food retrieval because candidate acceptability depends on relations that extend beyond text similarity.

Meal-planning and language-mediated systems operate at a different level. Stance4Health, reinforcement-learning planners, hybrid optimization, and SWITCHtoHEALTHY construct menus under personalized or expert-defined nutritional requirements [10,11,28,29]. F-RLP and ChatDiet use language-based interfaces for contextual food recommendation and personalized dietary guidance [30,31]. These systems produce or communicate dietary decisions, whereas recognition and nutrition-estimation methods supply food attributes that may support those decisions. Although these tasks are related, they are not equivalent to supervised candidate ranking in constrained food KBQA.

pFoodReQ formulates recipe recommendation as constrained KBQA through query expansion, knowledge-graph augmentation, constraint modeling, and recipe ranking [13]. D-pFoodReQ retains this food and constraint setting and examines positive-answer frequency within the ranker. This task distinction motivates the KBQA literature reviewed next.

### 2.2. Knowledge-Based Food Retrieval and Question Answering

Knowledge-base question answering follows two main routes. Semantic-parsing methods construct an executable representation of the question. Berant et al. learned logical forms from question–answer pairs, while Yih et al. generated query graphs through a staged search procedure [32,33]. Retrieval-based methods instead rank candidate entities from a local knowledge subgraph. Bordes et al. embedded questions and candidate subgraphs in a shared space, and later used memory networks to scale simple KBQA to large candidate sets [34,35]. BAMnet develops this retrieval route further. It represents each answer with type, relation-path, and context memories. Its bidirectional attention models both question-to-KB and KB-to-question interactions before answer scoring [14].

Recent KGQA research extends retrieval and reasoning to static, temporal, and multimodal knowledge graphs and increasingly combines graph retrieval with language models [36,37]. Think-on-Graph lets an LLM explore a knowledge graph as an external reasoning source, whereas UniKGQA unifies retrieval and reasoning for multi-hop question answering [38,39].

pFoodReQ instantiates the retrieval-based KBQA route on FoodKG and uses BAMnet to rank candidate recipes after query expansion, graph augmentation, and constraint modeling [13,14]. D-pFoodReQ preserves these structured memories and attention operations. It extends the answer ranker with a separate pathway that encodes answer frequencies observed in the training data.

### 2.3. Popularity Bias and Counterfactual Debiasing

In conventional recommender systems, popularity bias concerns items that receive disproportionate exposure or interaction because they are already frequently observed [15,16,17,40]. It is commonly measured from impressions, clicks, ratings, or user–item interactions. Unlike conventional item popularity, answer frequency in constrained KBQA records how often a candidate appears as a positive label in supervised training data. Both signals can favor repeatedly observed entities, but they arise from different data and should not be treated as equivalent.

Existing recommendation debiasing methods can be grouped by where they intervene. Output-level methods re-rank recommendations to increase long-tail exposure and balance accuracy with catalog representation [15,16]. Training-level methods alter sampled pairs or estimate propensities so that frequently exposed observations exert less influence [41,42,43,44]. Doubly robust estimation, conditional mutual-information minimization, and unbiased learning-to-rank theory further strengthen this objective-level line [45,46,47]. Model- and inference-level methods instead address popularity-related effects either in the prediction process or in learned representations. MACR formulates popularity debiasing through counterfactual reasoning [18], whereas PDA models popularity as a confounding factor and addresses its influence through causal intervention [19]. Together, these studies motivate treating popularity-related influences separately from relevance matching. DICE and AutoDebias learn disentangled representations or debiasing configurations [48,49,50,51]. These methods address exposure, interaction, or conformity signals in conventional recommendation settings [52]. D-pFoodReQ draws on this separation-and-intervention perspective in supervised constrained food KBQA, where the observable signal arises from repeated positive answer labels rather than user interactions. It characterizes this signal relative to the candidates available for each question, uses it in candidate scoring and question-level learning, and forms validation and test rankings without its direct score contribution.

Answer-side regularities have mainly been studied as shortcuts in supervised question answering. In visual question answering, language patterns may support predictions without sufficient visual grounding [53]. Related studies identify answer-frequency or option-selection regularities in visual question answering, multiple-choice tasks, and in-context learning [54,55,56]. Natural-language inference provides a related example, as annotation artifacts can make labels predictable without access to the complete input [57]. Taken together, these studies suggest that regularities in supervised labels may shape predictions alongside task-relevant evidence. We therefore examine the role of positive training-answer frequency in ranking, together with the query–candidate matching and constraint evidence specific to each question.

Taken together, prior recommender-system debiasing methods primarily target popularity arising from item exposure or user–item interactions [15,16,17,18,19,41,42,43,44,45,46,47,48,49,50,51,52]. Answer-frequency priors have also been investigated in other question-answering settings, particularly visual question answering [53,54,55,56]. To the best of our knowledge, the particular treatment considered here has not previously been examined in supervised constrained food KBQA: positive training-answer frequency is incorporated into candidate ranking and question-level learning during training, while validation and test rankings are formed without its direct contribution to candidate scores. This task-specific formulation constitutes the methodological contribution of D-pFoodReQ. The next section describes the design in detail.

## 3. Materials and Methods

### 3.1. Framework Overview

D-pFoodReQ retains the query expansion, candidate-generation procedure, knowledge-graph augmentation, constraint representation, and memory reasoning of pFoodReQ. Its modifications are confined to the answer-ranking stage. During training, BAMnet-D combines semantic and constraint evidence with an explicit score derived from positive training-answer frequency, while candidate-set-relative reweighting adjusts the contribution of each question to learning. During validation and testing, rankings are formed without the direct contribution of the explicit frequency term to candidate scores. The following sections define the frequency measure, its roles in candidate scoring and question-level learning, and the reference condition used for evaluation.

### 3.2. Task Formulation

Let $D={\left\{\left(q_{i},A_{i},Y_{i}\right)\right\}}_{i=1}^{N}$ denote the training set. Here, $q_{i}$ is a natural-language request. The set $A_{i}=\left\{a_{i1},\ldots ,a_{iC_{i}}\right\}$ contains the candidates from the upstream pFoodReQ modules. The set $Y_{i}\subseteq \left\{1,\ldots ,C_{i}\right\}$ contains the gold indices. Candidate $a_{ij}$ has a memory $M_{ij}$. The memory includes its entity type, relation paths, contextual entities, and constraint indicators. We use $X_{ij}$ for the query and candidate evidence. We use $m_{ij}\in \left\{0,1\right\}$ to mask padded candidates.

Let *h* index the BAMnet score outputs supervised during training. For candidate $a_{ij}$, the retained BAMnet pathway computes the score

(1) $$s_{q,ij}^{\left(h\right)}=f_{\theta }^{\left(h\right)}\left(X_{ij}\right).$$

This score summarizes the query–candidate evidence, including the constraint indicators contained in the candidate memory. The added answer-frequency value is not processed by this pathway. It is modeled separately and combined with $s_{q,ij}^{\left(h\right)}$ after the original BAMnet answer encoder.

### 3.3. Answer-Frequency Modeling

The benchmark provides supervised gold-answer labels but does not record impressions, clicks, ratings, or user–item interactions. We therefore define answer frequency from positive labels in the training split. This signal describes supervised positive-label support rather than exposure or interaction popularity. For answer entity *a*, the global training-answer frequency is

(2) $$n\left(a\right)=\sum_{i=1}^{N}\sum_{g\in Y_{i}}I\;\left[a_{ig}=a\right].$$

Validation and test labels are excluded. For each question, the counts are normalized by the largest count in its full candidate set

(3) $$p_{ij}=\frac{n(a_{ij})}{\max\;\left\{1,\max_{1\leq k\leq C_{i}}n\left(a_{ik}\right)\right\}},\;0\leq p_{ij}\leq 1.$$

The global count $n(a_{ij})$ is fixed across questions. In contrast, $p_{ij}$ also depends on the current candidate set. A candidate with zero training positive-label frequency has $p_{ij}=0$. The scalar is associated with candidate $a_{ij}$ but is not processed by the original BAMnet answer encoder. Instead, it enters the separate scoring branch defined below. The original entity, relation, context, and constraint representations are unchanged.

If no candidate for a question has appeared as a positive training answer, the denominator in Equation (3) is one, and the resulting frequency vector is zero. When all candidates have the same positive frequency, their normalized values are all one; the frequency term then changes their scores equally and leaves their relative ordering unchanged. The normalization is defined within each candidate set because ranking is performed among the alternatives available for the same question. It preserves their frequency ordering while placing the signal on a comparable scale across questions whose candidate pools differ substantially.

### 3.4. Frequency-Aware Additive Scoring

For candidate $a_{ij}$, the normalized training-answer frequency contributes the following term to its BAMnet score:

(4) $$s_{p,ij}=w_{p}p_{ij}.$$

The shared coefficient $w_{p}$ is learned jointly with the remaining BAMnet-D parameters. After padded entries have been masked, the combined score is

(5) $$S_{ij}^{\left(h\right)}=s_{q,ij}^{\left(h\right)}+m_{ij}s_{p,ij}.$$

With the observed frequency input, each supervised score used during training is

(6) $$S_{ij}^{\left(h,\mathrm{tr}\right)}=s_{q,ij}^{\left(h\right)}+m_{ij}s_{p,ij}=s_{q,ij}^{\left(h\right)}+m_{ij}w_{p}p_{ij}.$$

Because the frequency term contains no bias, a zero frequency input contributes zero to the score. Equation (6) makes its additive contribution explicit while the full ranker is optimized jointly.

### 3.5. Sample-Level Margin Loss

The original ranker uses a batch-reduced multi-label margin loss. D-pFoodReQ instead constructs an explicit loss for each question so that a question weight can be applied before batch averaging. All gold answers selected for a question enter this loss as positive candidates. The first position in their sampled ordering is used only to define the question-level weight below; it does not designate a uniquely correct or intrinsically more important answer. Let $C_{s}$ denote the length of the sampled candidate-score vector, $V_{i}$ its set of valid candidate indices, and $Y_{i}^{\prime }$ its selected gold indices. The loss for score output *h* is

(7) $$\begin{array}{cc} \ell_{i}^{\left(h\right)}= & \frac{1}{C_{s}}\sum_{g\in Y_{i}^{\prime }}\sum_{k\in V_{i}}I\left[k\neq g\right]\\ \; & \times \max\;\left\{0,\gamma -S_{ig}^{\left(h,\mathrm{tr}\right)}+S_{ik}^{\left(h,\mathrm{tr}\right)}\right\}. \end{array}$$

Here, γ=1. Padded candidates and targets do not contribute to the loss. For each selected gold answer, only its self-comparison is excluded. All other valid sampled candidates are retained. With multiple selected gold answers, this rule creates symmetric positive-positive terms. Their combined hinge penalty limits score differences larger than γ between selected gold answers.

### 3.6. Candidate-Set-Relative Loss Reweighting

The original pFoodReQ sampling procedure randomly orders the selected gold candidates before appending the sampled negatives. D-pFoodReQ follows this ordering and uses the normalized frequency $p_{i1}^{\prime }$ of the first sampled gold answer to define the weight of question *i*:

(8) $$\alpha_{i}=\frac{1}{\max\;\left\{p_{i1}^{\prime },0.01\right\}}.$$

Because candidate sampling is repeated during training, this position is not assigned permanently to a particular gold answer. The choice preserves the original sampling process without introducing a separate rule for selecting one correct answer as primary; all sampled gold answers remain positive candidates in Equation (7). The complete objective is

(9) $$L^{\left(h\right)}=\frac{1}{B}\sum_{i=1}^{B}\alpha_{i}\ell_{i}^{\left(h\right)},\;L=\frac{1}{H}\sum_{h=1}^{H}L^{\left(h\right)}.$$

Here, *B* is the batch size and *H* is the number of supervised BAMnet score outputs. Training counts range from 1 to 13, so the normalized frequency of the first sampled gold answer lies in [1/13,1] and the lower bound of 0.01 is not activated in the reported data. If all candidates have the same positive frequency, then $p_{i1}^{\prime }=1$ and $\alpha_{i}=1$. Questions otherwise receive larger weights when the first sampled gold answer has lower positive-label frequency relative to the most frequent candidate for that question. We refer to this procedure as sampled-first-gold, candidate-set-relative inverse-frequency reweighting. It follows the inverse-weighting principle used in propensity-based ranking [43], while $p_{i1}^{\prime }$ here denotes a normalized positive-label count.

### 3.7. Counterfactual Answer-Frequency Intervention

Guided by this separation-and-intervention perspective, we regard the explicitly modeled answer-frequency term as a distinct influence on candidate ranking. Training proceeds with the observed values of $P=\{p_{ij}\}$, allowing the model to account for the uneven positive-label support represented in the training data. Validation and test rankings are then formed under the reference condition do(P=0). The query representation, candidate memories, constraint evidence, and learned parameters remain unchanged, while the explicit frequency term no longer contributes directly to candidate scores:

(10) $$S_{ij}^{\left(h,\mathrm{cf}\right)}=S_{ij}^{\left(h\right)}{|}_{\mathrm{do}(P=0)}.$$

The frequency transformation has no bias term. Therefore,

(11) $$\mathrm{do}\left(P=0\right)\;⟹\;p_{ij}=0,\;s_{p,ij}=0,\;S_{ij}^{\left(h,\mathrm{cf}\right)}=s_{q,ij}^{\left(h\right)}.$$

For fixed $X_{ij}$ and learned parameters, the removed score is

(12) $$\Delta_{ij}^{\left(h,\mathrm{freq}\right)}=S_{ij}^{\left(h\right)}-S_{ij}^{\left(h,\mathrm{cf}\right)}=m_{ij}w_{p}p_{ij}.$$

Thus, for fixed query–candidate evidence and learned parameters, the counterfactual contrast in Equation (12) is the change in model score associated with the explicitly parameterized training-answer-frequency term. This contrast is defined with respect to the explicitly parameterized frequency term; frequency-associated regularities learned through the semantic pathway lie beyond this score-level intervention.

Candidates are ranked by the final counterfactual score $s_{ij}=S_{ij}^{\left(H,\mathrm{cf}\right)}$. Following pFoodReQ, the predicted answer set contains non-UNK candidates within margin τ of the highest score

(13) $${\hat{Y}}_{i}=\left\{a_{ij}\;|\;s_{ij}+\tau \geq \max_{k}s_{ik},\;a_{ij}\neq \mathrm{UNK}\right\}.$$

We use τ=0.9. The original constraint verification then checks candidate relation paths and answer types. D-pFoodReQ changes the ranker while preserving the upstream candidate and constraint pipeline.

In summary, D-pFoodReQ explicitly represents answer frequency during training and combines it with the retained BAMnet score. The question-level objective gives greater influence to samples whose first sampled gold answer has lower candidate-set-normalized frequency. Validation and test rankings are formed without the direct contribution of the explicit frequency term.

### 3.8. Experimental Design

We use the benchmark introduced with pFoodReQ [13]. It constructs constrained food-recommendation questions from FoodKG and health guidelines. The predefined data splits contain 4672 training questions, 1557 validation questions, and 2327 test questions. The training split contains 13,459 gold-answer labels covering 9650 distinct recipes, and each test question has 2.902 gold answers on average.

Candidate sets are produced by the unchanged upstream pFoodReQ pipeline and vary across questions. During training, dynamic candidate sampling is used with a memory capacity of 96, whereas evaluation is conducted over the complete valid candidate set generated for each question. Candidates that have not appeared as positive training answers are retained and ranked from their semantic and constraint evidence. Across the test set, candidate-set sizes range from 2 to 2646, with a mean of 475.426, a median of 344, and an interquartile range of 128–685. Their union contains 82,034 distinct candidates.

All methods use the same predefined data splits, FoodKG resources, candidate pools, vocabulary, upstream constraint processing, validation criterion, answer-set construction, and evaluation procedure. Answer-frequency counts are computed exclusively from positive labels in the training split.

Across the three complete runs, the order of the training examples was shuffled using NumPy random states initialized with seeds of 1234, 2345, and 3456. All other experimental settings were kept unchanged, and the results are reported as mean ± SD across the three runs. The models were implemented in Python 3.6 using NumPy 1.14.5, PyTorch 0.4.1, CUDA 10.0, and cuDNN 7.6. Experiments were conducted on Ubuntu 18.04. The main model and optimization settings are summarized in Table 1.

The numerical comparison is centered on methods evaluated within this common pFoodReQ ranking protocol. P-BOW and P-MatchNN retain their architecture-specific encoders, whereas all BAMnet-based methods share the upstream pFoodReQ pipeline. KERL is a relevant recent study that also reports the results on the pFoodReQ benchmark [26]. Its published evaluation, however, uses a different data split and forms recipe-name outputs by generating and aggregating predictions from serialized FoodKG contexts. Those results do not correspond to the common candidate-scoring and answer-selection protocol used here; directly importing them would combine differences in data, candidate handling, and output construction with the ranker comparison.

We compare the following baseline methods.

- **P-BOW and P-MatchNN** are the personalized bag-of-words and MatchNN baselines defined by pFoodReQ.
- **pFoodReQ** uses the original BAMnet ranking path and does not receive training-answer frequency.
- **pFoodReQ + Frequency (GoldPop)** adds the learned frequency score to the final hop of the original ranker without the D-pFoodReQ training objective or intervention.
- **D-pFoodReQ** uses BAMnet-D with the sample-level margin loss, candidate-set-relative question weighting, and rankings formed under P=0 during validation and testing.

Table 2 summarizes the loss formulation, frequency-based question weighting, and treatment of the explicit frequency-score branch across the compared configurations. The w/Original Loss variant restores the batch-reduced loss used by pFoodReQ, whereas w/o Reweighting retains the sample-level margin loss but assigns equal weight to all questions. Reweighting-only retains the sample-level loss and frequency-based question weighting without introducing an explicit frequency-score branch, while w/o Intervention retains the observed frequency input when validation and test rankings are formed.

### 3.9. Evaluation Measures

We organize the measures into three groups.

*Answer quality and ranking.* Question-level F1 is used as the primary measure of returned answer-set quality because each question may have multiple gold answers; the remaining measures provide complementary assessments of candidate ranking. Precision is computed from the complete returned answer set for each question and then macro-averaged over all test questions. Mean reciprocal rank (MRR), mean average precision (MAP), mean average recall (MAR), Recall@5, and normalized discounted cumulative gain at five (NDCG@5) evaluate candidate ordering. Recall@5 denotes recall at five. NDCG discounts correct answers at lower ranks [58]. Following the original pFoodReQ implementation, MAP and MAR are computed at a cutoff of 10; for consistency with the terminology used in that work, we retain the labels MAP and MAR in the following tables.

Results are summarized as the mean and standard deviation across the three complete runs. Statistical uncertainty in the comparison between D-pFoodReQ and pFoodReQ was assessed by paired bootstrap resampling for F1, MRR, MAP, MAR, Recall@5, and NDCG@5. For each measure, question-level scores were first averaged across the three runs for each model. The resulting paired differences over the 2327 test questions were then resampled 10,000 times. We report the observed mean difference, its two-sided 95% percentile confidence interval (CI), and the corresponding empirical *p* value obtained with a finite-sample correction.

*Constraint violations.* Knowledge-based and hybrid recommender systems provide a general framework for incorporating domain knowledge and explicit user requirements into recommendation [59]. In the constrained food-retrieval setting, we define Viol@K as a diagnostic of forbidden-ingredient compliance. Its task-specific definition follows the ingredient-constraint setting of pFoodReQ and recent constraint-satisfaction evaluation in diet-aware recipe retrieval [13,60]. Let $Q_{c}$ be the set of test questions that contain at least one explicit forbidden-ingredient constraint. For question *i*, let $F_{i}$ be its forbidden-ingredient set, let $R_{i}^{\leq K}$ contain up to the first *K* returned recipes, and let G(r) be the ingredient set of recipe *r*. We define

(14) $$v_{i}^{\left(K\right)}=\left\{\begin{array}{cc} \frac{1}{|R_{i}^{\leq K}|}\sum_{r\in R_{i}^{\leq K}}I\left[F_{i}\cap G\left(r\right)\neq ⌀\right],\; & |R_{i}^{\leq K}|>0,\\ 0, & |R_{i}^{\leq K}|=0, \end{array}\right.$$

and

(15) $$\mathrm{Viol}@K=\frac{1}{|Q_{c}|}\sum_{i\in Q_{c}}v_{i}^{\left(K\right)}.$$

Questions without an explicit forbidden-ingredient constraint are excluded. A returned recipe is considered to violate the constraint if its ingredient set contains at least one forbidden ingredient specified in the query.

*Frequency and catalog diagnostics.* For the following catalog diagnostics, *N* denotes the number of test questions. We define the head set as recipes that appeared at least twice as positive answers in the training set. The remaining recipes form the tail set T. This definition yields 2797 head recipes among the 9650 recipes observed as positive training answers. For each test question, let

(16) $$t_{i}=\left\{\begin{array}{cc} \frac{\left|{\hat{Y}}_{i}\cap T\right|}{\left|{\hat{Y}}_{i}\right|}, & |{\hat{Y}}_{i}|>0,\\ 0, & |{\hat{Y}}_{i}|=0. \end{array}\right.$$

TailCov is the macro-average

(17) $$\mathrm{TailCov}=\frac{1}{N}\sum_{i=1}^{N}t_{i}.$$

This macro-average gives every test question equal weight. TailCov measures the tail composition of returned answer sets [61].

Coverage@10 measures aggregate catalog coverage. Let $C_{\mathrm{test}}$ be the union of the full candidate sets for all test questions. We define

(18) $$\mathrm{Coverage}@10=\frac{\left|{⋃}_{i=1}^{N}{\hat{Y}}_{i}^{\leq 10}\right|}{|C_{\mathrm{test}}|},$$

where ${\hat{Y}}_{i}^{\leq 10}$ contains at most ten returned recipes. In this benchmark, $|C_{\mathrm{test}}|=82,034$. Every returned recipe belongs to this candidate union. Coverage complements ranking accuracy by measuring how broadly a system uses the available catalog [61,62,63]. We also report F1 across training-answer-frequency regimes. Finally, we examine the frequency composition of predicted answer sets.

## 4. Results

### 4.1. Baseline Comparison

Table 3 compares all baseline methods under the common experimental protocol described above.

Across the three runs, D-pFoodReQ attained the highest mean value for all six measures in Table 3. Its F1 was 0.6296±0.0206, compared with 0.6133±0.0227 for pFoodReQ. MAP and MAR increased from 0.6106±0.0246 and 0.6012±0.0243 to 0.6650±0.0054 and 0.6538±0.0066, respectively, and the same direction was observed for MRR, Recall@5, and NDCG@5. Paired question-level bootstrap analyses supported these differences: the observed mean differences were 0.0163 for F1 (95% CI, 0.0080–0.0243), 0.0339 for MRR (0.0258–0.0421), 0.0545 for MAP (0.0466–0.0623), 0.0526 for MAR (0.0450–0.0604), 0.0746 for Recall@5 (0.0654–0.0840), and 0.0531 for NDCG@5 (0.0450–0.0611). The empirical *p* value was 0.0002 for each comparison.

GoldPop yielded a lower mean F1 than pFoodReQ and showed substantially greater variation across runs. Although its mean MRR, MAP, MAR, Recall@5, and NDCG@5 values were slightly higher than those of pFoodReQ, these differences were small relative to the run-to-run variation and were not accompanied by stronger answer-set F1. Thus, directly adding a frequency score to the original ranker did not yield a consistent improvement over pFoodReQ under the same experimental protocol. The following analysis examines the method components in greater detail.

### 4.2. Ablation Analysis

Table 4 presents the results for the component configurations summarized in Table 2, all evaluated under the same experimental conditions.

D-pFoodReQ achieved the highest mean value for all six measures. The w/Original Loss variant came closest in F1 (0.6210±0.0213 versus 0.6296±0.0206) but remained below D-pFoodReQ on all five ranking measures. Removing frequency-based question weighting led to lower mean F1, MAP, and MAR, despite a comparatively strong Recall@5.

The other configurations revealed a different balance between answer-set and ranking quality. Reweighting-only obtained the second-highest mean values on all five ranking measures, but its F1 (0.5921±0.0282) remained below both pFoodReQ and D-pFoodReQ. Retaining the observed frequency input during validation and testing in w/o Intervention produced lower mean values than D-pFoodReQ across all six measures. Taken together, no partial configuration matched the full model’s balance of F1 and ranking quality. These comparisons describe the behavior of complete, independently trained configurations under the same experimental protocol.

### 4.3. Sensitivity to Multiple-Gold Weighting

The default question weight uses the first gold answer in the dynamic sampled ordering. We assessed the sensitivity of this choice by replacing its normalized frequency with the mean normalized frequency of all gold answers; all other settings were unchanged. Table 5 compares the sampled-first-gold and Mean-gold weighting strategies.

Mean-gold did not provide a consistent improvement: its three-run means were lower and its variation was greater for F1, MAP, and MAR. We therefore retained sampled-first-gold weighting as the default implementation. This sensitivity analysis also shows that the aggregation of multiple gold-answer frequencies is a consequential modeling choice rather than a purely notational detail.

Table 6 places F1 alongside constraint and catalog diagnostics so that these complementary results can be interpreted together.

Across the three runs, D-pFoodReQ achieved lower mean Viol@1, Viol@3, and Viol@5 values than pFoodReQ, indicating better average performance on the benchmark’s explicit forbidden-ingredient constraint diagnostic at all reported cutoffs. Because the absolute violation rates were low for both methods, these results are interpreted specifically in terms of the constraints measured by Viol@K.

D-pFoodReQ used more of the available candidate catalog than pFoodReQ, with mean Coverage@10 values of 0.0734±0.0051 and 0.0570±0.0015, respectively, while their TailCov values remained close. Reweighting-only obtained the highest Coverage@10, and w/o Reweighting obtained the highest TailCov, but neither matched the F1 of D-pFoodReQ. Because Coverage@10 reflects the breadth of catalog use and varies with the composition and size of the returned answer sets, we interpret it as a complementary diagnostic rather than a substitute for answer-quality measures.

### 4.4. Generalization Across Training-Answer-Frequency Regimes

We next test whether the improvement extends beyond answers that repeatedly occur as positive training labels. Gold strings are lowercased and trimmed. They are then resolved through the available name-to-entity map. For test question *i*, let $G_{i}$ contain its gold-answer strings. Let M(g) be the set of matched training entities. Let $n_{\mathrm{train}}\left(u\right)$ be the positive-label count of entity *u*. We define

(19) $$\tilde{n}\left(g\right)=\left\{\begin{array}{cc} \max_{u\in M(g)}n_{\mathrm{train}}\left(u\right), & M(g)\neq ⌀,\\ 0, & M(g)=⌀, \end{array}\right.\;P_{i}=\max_{g\in G_{i}}\tilde{n}\left(g\right).$$

The maximum assigns $P_{i}=0$ only when none of the gold answers appeared as a positive training label. We call this the zero-training-answer-frequency subset. Bucket membership depends only on the gold answers. Every method is therefore evaluated on the same questions.

The four buckets contain 1355, 751, 188, and 33 test questions, corresponding to 58.2%, 32.3%, 8.1%, and 1.4% of the test set, respectively. Figure 3 shows this distribution.

Table 7 reports question-level F1 for the same four regimes.

Averaged over the three runs, D-pFoodReQ and pFoodReQ attained nearly identical mean F1 in the largest $P_{i}=0$ subset (0.6251±0.0205 and 0.6253±0.0257, respectively). D-pFoodReQ achieved higher mean F1 in each nonzero-frequency regime: 0.6299±0.0248, 0.6558±0.0074, and 0.6599±0.0277 for $P_{i}=1$–2, 3–5, and $P_{i}\geq 6$, compared with 0.5900±0.0209, 0.6337±0.0239, and 0.5376±0.0236 for pFoodReQ. Within the component comparison, w/Original Loss yielded the highest mean F1 in the zero-frequency subset, whereas D-pFoodReQ achieved the highest mean F1 in all three nonzero-frequency regimes. Thus, the overall improvement over pFoodReQ was associated with gains in the nonzero-frequency groups, while the two models attained nearly identical mean answer-set F1 on questions whose gold answers had not appeared as positive training labels. The $P_{i}\geq 6$ group comprised only 33 questions (1.4% of the test set); the groupwise results therefore do not establish a monotonic relation between training-answer frequency and the magnitude of the improvement.

Table 8 compares ranking quality on the zero-training-answer-frequency subset with the corresponding results for all test questions.

The zero-frequency subset also provides a useful setting for examining the respective roles of the modified training objective and the explicit frequency-score branch. On this subset, D-pFoodReQ attained a mean Recall@5 of 0.7312±0.0059 and a mean NDCG@5 of 0.7010±0.0069, compared with 0.6711±0.0281 and 0.6628±0.0272, respectively, for pFoodReQ. Their mean F1 values were nearly identical (0.6251±0.0205 and 0.6253±0.0257). Thus, on questions whose gold answers had no positive training frequency, D-pFoodReQ attained a mean answer-set F1 nearly identical to that of pFoodReQ while achieving higher Recall@5 and NDCG@5.

Reweighting-only yielded mean Recall@5 and NDCG@5 values close to those of D-pFoodReQ (0.7309±0.0245 versus 0.7312±0.0059 and 0.6989±0.0207 versus 0.7010±0.0069, respectively), but achieved a lower mean F1 (0.5898±0.0243 versus 0.6251±0.0205). Because none of the gold answers in this subset appeared as a positive training label, these gains cannot be attributed to a positive training-answer-frequency value for the gold answers themselves. Taken together, these comparisons suggest that the modified training objective plays an important role in top-five ranking on zero-frequency questions. Compared with Reweighting-only, the complete D-pFoodReQ configuration achieved comparable mean Recall@5 and NDCG@5 while yielding a higher mean answer-set F1.

### 4.5. Frequency Composition of Predicted Answer Sets

Finally, we examine whether explicit frequency modeling changes the composition of predicted answer sets. Each predicted recipe is assigned to one of the four training-answer-frequency regimes. For regime *b*, the output share is

(20) $$R_{b}=\frac{\sum_{i}\sum_{r\in {\hat{Y}}_{i}}I\left[n_{\mathrm{train}}\left(r\right)\in b\right]}{\sum_{i}\left|{\hat{Y}}_{i}\right|}.$$

Every returned answer contributes once, regardless of rank. The models produce answer sets of different sizes. Each share is therefore normalized by the total number of answers returned by the same model. Table 9 reports these relative output compositions together with Precision and F1. Each frequency share is independently rounded to two decimal places. The frequency shares describe output composition and have no preferred direction.

Here, a positive-frequency recipe is one that appeared at least once as a gold answer in the training split; this designation reflects supervised label recurrence rather than real-world popularity derived from user exposure or interaction. Despite its higher mean F1, D-pFoodReQ produced an output-frequency distribution close to that of pFoodReQ. Recipes with positive training-answer frequency accounted for 21.66±2.00% and 21.25±1.06% of their returned answers, respectively. Thus, the improvement was achieved without a marked redistribution of predictions toward recipes that recurred as positive labels in training.

GoldPop and w/o Intervention returned larger positive-frequency shares, averaging 27.43±6.80% and 33.47±2.13%, respectively, while yielding lower answer-set F1 than their corresponding comparators. Reweighting-only produced the smallest positive-frequency share among the configurations considered but also obtained lower F1 than D-pFoodReQ. These descriptive results show no simple relationship between output-frequency composition and answer-set quality. Rather, D-pFoodReQ improves F1 while retaining an output-frequency profile similar to that of its parent model.

Taken together, these findings are consistent with the intervention design described in Section 3.7, in which training-answer frequency informs model learning, whereas the explicit frequency term is omitted from the candidate scores used to form validation and test rankings.

## 5. Discussion

Reliable recipe retrieval is an upstream requirement for nutrition-aware food recommendation: structured knowledge about ingredients, nutrients, and dietary constraints can guide a user only when suitable candidates remain competitive in the answer ranker. In the three-run evaluation under the same experimental protocol, D-pFoodReQ achieved higher mean answer-set quality and rank-sensitive performance than pFoodReQ. The mean F1 advantage was modest but positive, whereas the gains in MRR, MAP, MAR, Recall@5, and NDCG@5 were larger. The paired question-level bootstrap confidence intervals for all six measures remained above zero, complementing the variation observed across complete runs.

The component results reveal a distinction between ranking quality and the balance of the returned answer set. Reweighting-only produced strong rank-sensitive results but did not improve mean F1 over pFoodReQ. D-pFoodReQ retained this ranking advantage while obtaining the highest mean F1. Conversely, the w/o Intervention configuration retained the explicit frequency score and returned a substantially larger proportion of recipes with positive training frequency, together with lower average performance. These comparisons support complementary roles for the modified objective and the treatment of the explicit frequency signal, while the separately trained variants do not imply a strictly additive decomposition of their effects.

The frequency-regime analysis showed that D-pFoodReQ attained a mean answer-set F1 nearly identical to that of pFoodReQ and improved top-five ranking quality on the large zero-frequency subset. Its F1 gains over pFoodReQ occurred in all three nonzero-frequency groups. w/Original Loss achieved a slightly higher zero-frequency F1, and Reweighting-only approached the full model on the two ranking measures. Mean violation rates were lower than those of pFoodReQ at all three reported cutoffs, although their small magnitudes and between-run variation do not support a broad claim of uniformly stronger constraint handling. Likewise, broader Coverage@10 accompanied changes in the returned answer sets and is best read as a secondary catalog-use characteristic rather than as a direct measure of accuracy or fairness.

Positive training-answer frequency is not intrinsically uninformative. Repeated labels may reflect recurring recipe types, annotation coverage, or genuine regularities in the benchmark. The relevant concern is whether this query-independent signal receives sufficient influence to displace semantic and constraint evidence specific to a new question. The present results favor using frequency to inform learning while withholding its direct additive contribution when validation and test rankings are formed. The Mean-gold analysis also indicates that the way frequency is summarized across multiple correct answers can affect the outcome, providing a basis for studying alternative weighting rules.

The scope of these findings is the controlled experimental setting considered here, in which all methods share the same FoodKG resources, candidate-generation procedure, constraint processing, and answer-selection protocol. This design supports a focused comparison of answer-frequency treatment within the ranker. Because the benchmark does not contain user-interaction records, the present analysis concerns training-answer frequency rather than user-derived popularity. The explicitly parameterized frequency pathway is the focus here; examining how frequency-associated regularities are represented in the jointly learned semantic space provides an important direction for future work. Future studies will assess transfer across other datasets, candidate generators, and ranking architectures, together with aligned comparisons to recent end-to-end food-recommendation systems. Finally, the present Viol@K analysis concerns forbidden-ingredient annotations; nutritional adequacy, health outcomes, and user satisfaction remain distinct questions for future food-recommendation research [3,4,5,29].

## 6. Conclusions

We presented D-pFoodReQ for answer-frequency-aware ranking in constrained food KBQA. The method retains the FoodKG and constraint-processing pipeline of pFoodReQ while introducing candidate-set-relative frequency modeling, question-level loss reweighting, and validation and test rankings formed without the direct contribution of the explicit frequency score. Across three complete runs conducted under the same experimental protocol, D-pFoodReQ achieved higher mean F1, MRR, MAP, MAR, Recall@5, and NDCG@5 than pFoodReQ. On questions whose gold answers had zero positive training frequency, it attained a mean answer-set F1 nearly identical to that of pFoodReQ while achieving higher Recall@5 and NDCG@5. The component analyses further showed that Reweighting-only reproduced much of the top-five ranking gain, whereas the complete D-pFoodReQ configuration achieved higher answer-set F1 than Reweighting-only. These findings support D-pFoodReQ as an effective extension of the pFoodReQ answer ranker for the experimental setting considered here. Future work will examine its transfer across datasets and candidate-generation procedures and relate training-answer frequency to richer interaction and nutritional evidence.

## Figures and Tables

**Figure 1:**
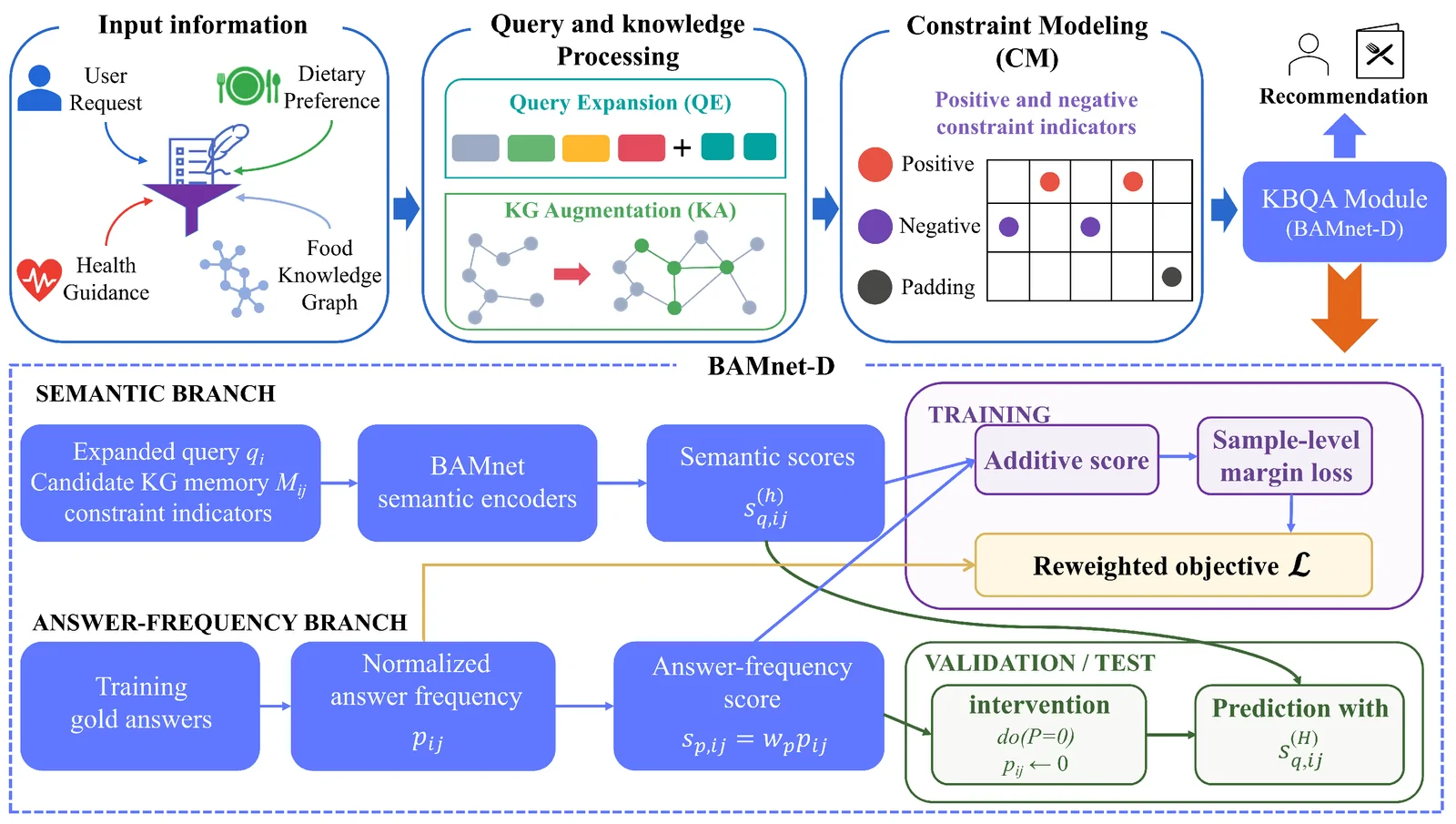
Overview of D-pFoodReQ. The original pFoodReQ pipeline supplies query, candidate-memory, and constraint evidence to BAMnet-D. During training, candidate scores incorporate normalized positive training-answer frequency and the question-level objective is reweighted accordingly. Validation and test rankings are formed under P=0, so that the explicit frequency term makes no direct contribution to the returned scores. KG, knowledge graph; KBQA, knowledge-base question answering; BAMnet, bidirectional attentive memory network.

**Figure 2:**
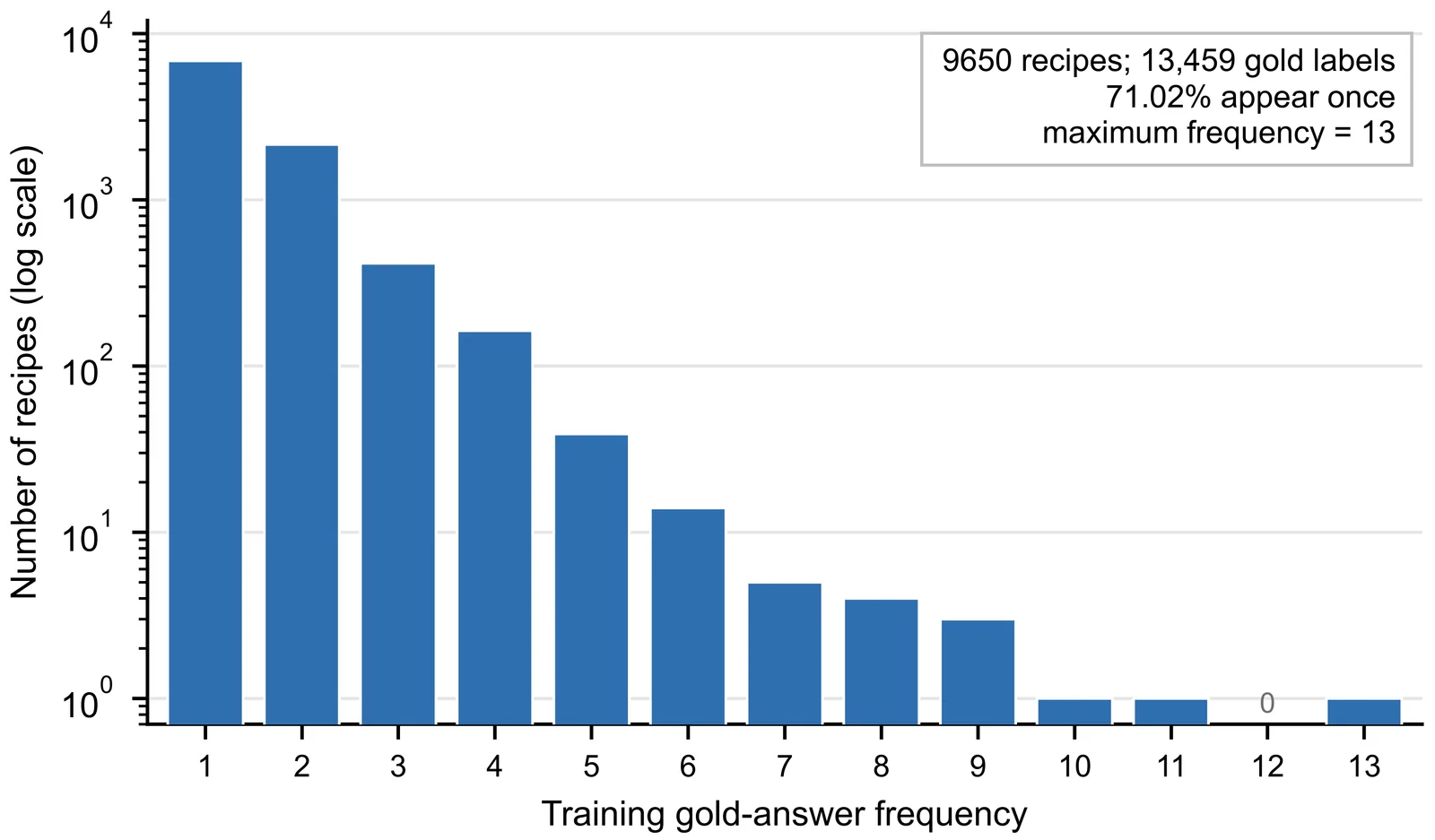
Distribution of positive training-answer frequency. The vertical axis uses a logarithmic scale. Among 9650 distinct gold answers, 6853 occur once. The maximum observed frequency is 13.

**Figure 3:**
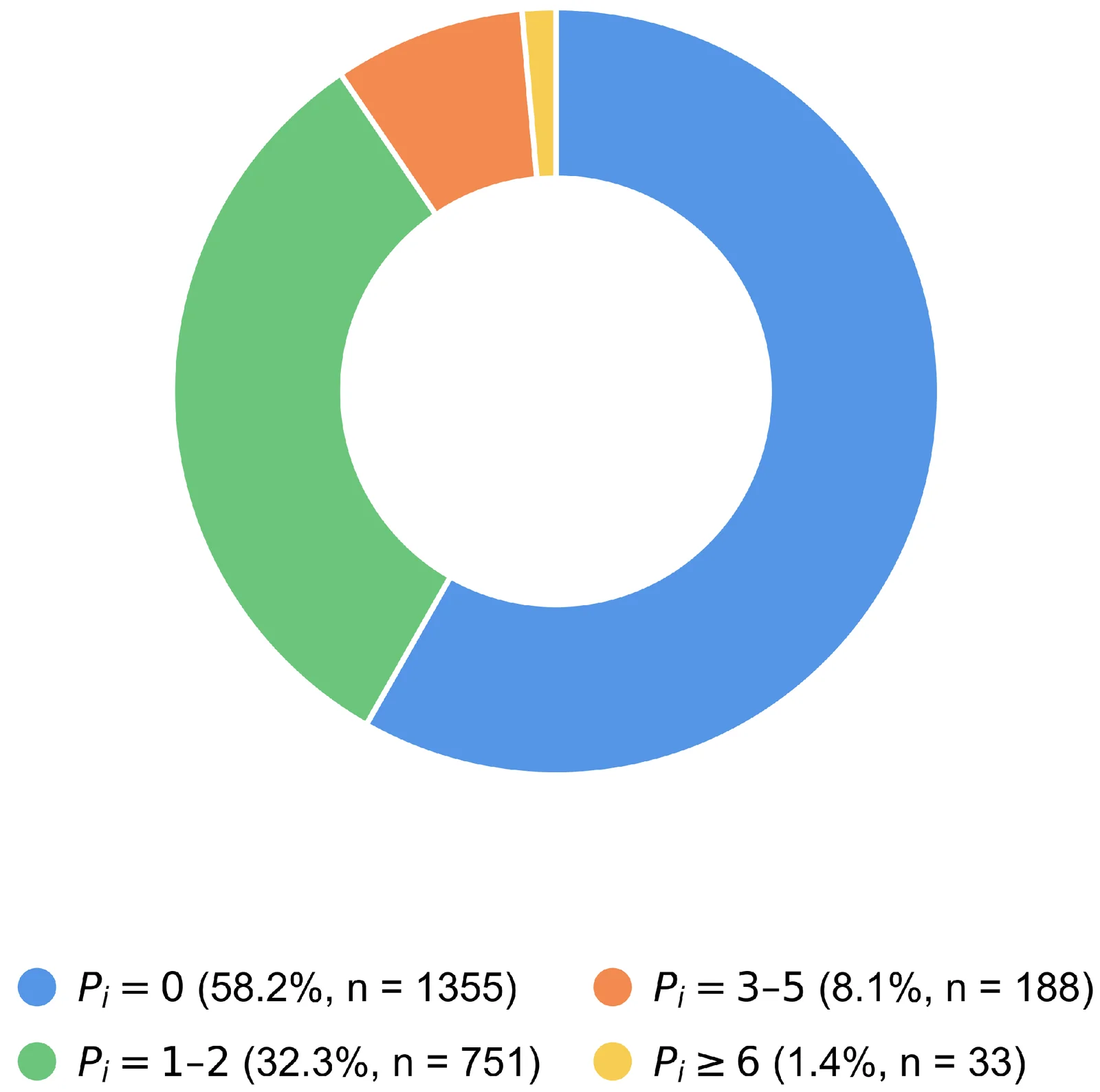
Distribution of the 2327 test questions under the maximum-gold-frequency rule in Equation (19). The $P_{i}=0$ bucket contains questions for which no gold answer appeared as a positive training label.

**Table 1:** Main model and optimization settings.

Setting	Value
Embeddings and model dimensions	300-dimensional GloVe 840B, updated during training; hidden/output dimensions, 128/128; constraint-mark embedding dimension, 40
Memory configuration	One hop; training memory size, 96
Word/query/answer dropout	0.3/0.3/0.2
Optimizer, initial learning rate, and gradient clipping	Adam; 0.001; maximum gradient norm, 5.0
Batch size and gradient accumulation	16; 2 steps
Training duration and learning-rate schedule	Maximum 100 epochs; early-stopping patience, 20 epochs; ReduceLROnPlateau on validation F1, patience 6
Loss and weighting settings	Margin =1.0; question-level weight =1/max{p,0.01}, where *p* is the normalized frequency of the first sampled gold answer
Prediction and model selection	Prediction margin, 0.9; highest validation F1
Frequency input and normalization	Positive-answer counts from the training split, normalized by the maximum count in each candidate set
Frequency-score component	One shared coefficient $w_{p}$; no bias or activation; initialized uniformly over [−1,1]
Training-data shuffle seeds	1234, 2345, and 3456

**Table 2:** Method components in the ablation analysis.

Method	Loss Formulation	Frequency-Based Question Weighting	Frequency-Score Branch in Training	Frequency Input at Validation/Test
pFoodReQ	Original loss	No	No	N/A
w/Original Loss	Original loss	No	Yes	P=0
w/o Reweighting	Sample-level margin loss	No	Yes	P=0
Reweighting-only	Sample-level margin loss	Yes	No	N/A
w/o Intervention	Sample-level margin loss	Yes	Yes	Observed *P*
D-pFoodReQ	Sample-level margin loss	Yes	Yes	P=0

Original loss denotes the batch-reduced MultiLabelMarginLoss used by pFoodReQ. P=0 indicates that the explicit frequency input is set to zero when validation and test rankings are formed, whereas Observed *P* indicates that this input is retained. N/A indicates that the method contains no explicit frequency-score branch.

**Table 3:** Baseline results under the common experimental protocol (mean ± SD over three runs).

Method	F1	MRR	MAP	MAR	Recall@5	NDCG@5
P-BOW	0.0322±0.0007	0.0461±0.0016	0.0368±0.0010	0.0366±0.0010	0.0488±0.0019	0.0415±0.0013
P-MatchNN	0.4260±0.0049	0.5041±0.0028	0.4506±0.0034	0.4481±0.0034	0.4870±0.0051	0.4740±0.0033
pFoodReQ	0.6133±0.0227	0.6997±0.0244	0.6106±0.0246	0.6012±0.0243	0.6377±0.0252	0.6471±0.0250
GoldPop	0.5939±0.0583	0.7109±0.0341	0.6233±0.0294	0.6133±0.0286	0.6553±0.0325	0.6608±0.0312
D-pFoodReQ	0.6296±0.0206	0.7336±0.0039	0.6650±0.0054	0.6538±0.0066	0.7123±0.0071	0.7003±0.0040

Bold and underlined values indicate the best and second-best mean results in each column, respectively.

**Table 4:** Ablation results for answer quality and ranking (mean ± SD over three runs).

Method	F1	MRR	MAP	MAR	Recall@5	NDCG@5
pFoodReQ	0.6133±0.0227	0.6997±0.0244	0.6106±0.0246	0.6012±0.0243	0.6377±0.0252	0.6471±0.0250
w/Original Loss	0.6210±0.0213	0.7136±0.0326	0.6230±0.0266	0.6131±0.0258	0.6495±0.0245	0.6600±0.0285
w/o Reweighting	0.5539±0.0214	0.6675±0.0403	0.6084±0.0461	0.6002±0.0453	0.6666±0.0576	0.6409±0.0477
w/o Intervention	0.5581±0.0361	0.6623±0.0580	0.5849±0.0568	0.5765±0.0542	0.6312±0.0624	0.6215±0.0601
Reweighting-only	0.5921±0.0282	0.7242±0.0199	0.6534±0.0187	0.6437±0.0180	0.7030±0.0236	0.6898±0.0205
D-pFoodReQ	0.6296±0.0206	0.7336±0.0039	0.6650±0.0054	0.6538±0.0066	0.7123±0.0071	0.7003±0.0040

Bold and underlined values indicate the highest and second-highest mean results in each column, respectively.

**Table 5:** Sensitivity to the aggregation of multiple gold-answer frequencies (mean ± SD over three runs).

Weighting Strategy	F1	MAP	MAR
Sampled-first-gold (D-pFoodReQ)	0.6296±0.0206	0.6650±0.0054	0.6538±0.0066
Mean-gold	0.5993±0.0457	0.6468±0.0778	0.6367±0.0748

The sampled-first-gold strategy is the default used in D-pFoodReQ. Bold values indicate the higher mean result in each column.

**Table 6:** Answer quality, constraint violations, and catalog-use diagnostics (mean ± SD over three runs).

	Answer Quality	Constraint Violations			Catalog Use	
Method	F1↑	Viol@1↓	Viol@3↓	Viol@5↓	TailCov↑	Coverage@10↑
pFoodReQ	0.6133±0.0227	0.0066±0.0025	0.0074±0.0028	0.0076±0.0029	0.8795±0.0017	0.0570±0.0015
w/Original Loss	0.6210±0.0213	0.0049±0.0015	0.0055±0.0014	0.0059±0.0014	0.8799±0.0035	0.0575±0.0007
w/o Reweighting	0.5539±0.0214	0.0059±0.0039	0.0072±0.0048	0.0081±0.0046	0.8870±0.0034	0.0795±0.0060
w/o Intervention	0.5581±0.0361	0.0099±0.0027	0.0112±0.0036	0.0121±0.0031	0.8190±0.0167	0.0652±0.0061
Reweighting-only	0.5921±0.0282	0.0085±0.0037	0.0111±0.0054	0.0121±0.0053	0.8789±0.0013	0.0848±0.0084
D-pFoodReQ	0.6296±0.0206	0.0039±0.0009	0.0054±0.0011	0.0065±0.0013	0.8810±0.0036	0.0734±0.0051

Upward and downward arrows indicate whether higher or lower values are preferred. Bold and underlined values indicate the best and second-best mean results in each column, respectively.

**Table 7:** Question-Level F1 across training-answer-frequency regimes (mean ± SD over three runs).

Method	0 (*n* = 1355)	1–2 (*n* = 751)	3–5 (*n* = 188)	≥6 (*n* = 33)	Overall
pFoodReQ	0.6253±0.0257	0.5900±0.0209	0.6337±0.0239	0.5376±0.0236	0.6133±0.0227
GoldPop	0.5993±0.0572	0.5772±0.0609	0.6239±0.0633	0.5825±0.0480	0.5939±0.0583
w/ Original Loss	0.6343±0.0228	0.5969±0.0212	0.6317±0.0142	0.5631±0.0203	0.6210±0.0213
w/o Reweighting	0.5533±0.0168	0.5480±0.0282	0.5753±0.0295	0.5872±0.0255	0.5539±0.0214
w/o Intervention	0.5472±0.0323	0.5596±0.0360	0.6271±0.0631	0.5740±0.0567	0.5581±0.0361
Reweighting-only	0.5898±0.0243	0.5952±0.0274	0.5998±0.0624	0.5694±0.0546	0.5921±0.0282
D-pFoodReQ	0.6251±0.0205	0.6299±0.0248	0.6558±0.0074	0.6599±0.0277	0.6296±0.0206

The columns headed 0, 1–2, 3–5, and ≥6 denote the corresponding $P_{i}$ regimes. Bold and underlined values indicate the highest and second-highest mean results in each column, respectively.

**Table 8:** Zero-training-answer-frequency and overall ranking results (mean ± SD over three runs).

Method	Recall@5		NDCG@5	
	Zero-Frequency Questions	All Questions	Zero-Frequency Questions	All Questions
pFoodReQ	0.6711±0.0281	0.6377±0.0252	0.6628±0.0272	0.6471±0.0250
GoldPop	0.6794±0.0298	0.6553±0.0325	0.6622±0.0293	0.6608±0.0312
w/ Original Loss	0.6819±0.0255	0.6495±0.0245	0.6729±0.0284	0.6600±0.0285
w/o Reweighting	0.6889±0.0599	0.6666±0.0576	0.6487±0.0473	0.6409±0.0477
w/o Intervention	0.6439±0.0641	0.6312±0.0624	0.6111±0.0583	0.6215±0.0601
Reweighting-only	0.7309±0.0245	0.7030±0.0236	0.6989±0.0207	0.6898±0.0205
D-pFoodReQ	0.7312±0.0059	0.7123±0.0071	0.7010±0.0069	0.7003±0.0040

Zero-Frequency Questions denotes the 1355 test questions for which no gold answer appeared as a positive training label. All Questions denotes the complete test set. Recall@5, recall at 5; NDCG@5, normalized discounted cumulative gain at 5. Bold and underlined values indicate the best and second-best mean results in each column, respectively.

**Table 9:** Returned-recipe frequency distribution with precision and F1 (mean ± SD over three runs).

Method	Returned-Recipe Frequency Distribution (%)				Precision	F1
	0	1–2	3–5	≥6		
pFoodReQ	78.75±1.06	18.12±0.93	2.85±0.14	0.28±0.05	0.6378±0.0203	0.6133±0.0227
GoldPop	72.57±6.80	22.88±5.44	4.12±1.25	0.44±0.14	0.6072±0.0752	0.5939±0.0583
w/o Intervention	66.53±2.13	26.36±0.93	6.36±1.32	0.75±0.11	0.5570±0.0239	0.5581±0.0361
Reweighting-only	79.61±1.96	17.77±1.56	2.38±0.35	0.24±0.05	0.5709±0.0292	0.5921±0.0282
D-pFoodReQ	78.34±2.00	18.76±1.65	2.63±0.31	0.27±0.07	0.6110±0.0207	0.6296±0.0206

The four distribution columns group returned recipes by their positive-label frequencies in training. Each row is normalized over all recipes returned by the corresponding method. Precision and F1 are computed from the complete returned answer sets. Bold and underlined values indicate the best and second-best mean results in the Precision and F1 columns, respectively. The frequency-distribution columns are descriptive and have no preferred direction.

## Data Availability

The pFoodReQ benchmark used in this study is described in Reference [13]. The source code, model configurations, and evaluation scripts supporting the reported analyses are publicly available at https://github.com/bishi17/D-pFoodreq (accessed on 23 August 2026).

## Abbreviations

The following abbreviations are used in this manuscript.

BAMnet	Bidirectional attentive memory network
CI	Confidence interval
KBQA	Knowledge-base question answering
KG	Knowledge graph
MRR	Mean reciprocal rank
MAP	Mean average precision
MAR	Mean average recall
NDCG	Normalized discounted cumulative gain
